# Predictors of deliberate self-harm among adolescents: Answers from a cross-sectional study on India

**DOI:** 10.1186/s40359-021-00705-4

**Published:** 2021-12-18

**Authors:** Debashree Sinha, Shobhit Srivastava, Prem Shankar Mishra, Pradeep Kumar

**Affiliations:** 1 Department of Development Studies, International Institute for Population Sciences, Mumbai, Maharashtra 400088 India; 2 Department of Research and Innovation, MAMTA Health Institute for Mother and Child, New Delhi, New Delhi 110048 India; 3 Department of Population Research Centre, Institute for Social and Economic Change, Bengaluru, Karnataka 560072 India; 4 Population Council India Office, New Delhi, 110003 India

**Keywords:** Deliberate self-harm, Adolescents, Internet access, Parental physical abuse, Depressive symptoms, UDAYA

## Abstract

**Background:**

Although existing research supports the correlation of hereditary and psychological factors with an adolescent’s deliberate self-harm, there is a dearth of research that focus on their socio-economic characteristics. This paper intends to identity the potential risk factors that influence an adolescent’s deliberate self-harm.

**Methods:**

Data for this study was obtained from Understanding the Lives of Adolescents and Young Adults (UDAYA) study conducted in 2015–16 with sample of 5,969 adolescent boys and 9,419 girls aged 10–19 years. The outcome variable was deliberate self-harm among adolescents. The explanatory variables added in the study were age, current schooling status, working status, media exposure, access to internet, parental abuse, involvement in fights, substance use, depressive symptoms, caste, religion, wealth index, residence and states. Bivariate analysis along with binary logistic regression analysis was done to fulfill the study objectives.

**Results:**

About 4.5% and 3.2% of adolescent boys and girls, respectively had deliberate self-harm. The odds of deliberate self-harm were 50 per cent more likely among adolescent girls who had internet access [OR 1.50; CI 1.05–2.16]. The likelihood of deliberate self-harm was 49 per cent and 61 per cent significantly more likely among adolescent boys [OR 1.49; CI 1.11–2.0] and girls [OR 1.61; CI 1.27–2.04] who experienced parental physical abuse respectively. With reference to minimal/mild depressive symptoms, adolescents who had moderate [boys-OR 2.10; CI 1.29–3.4 and girls-OR 2.50; CI 1.774–3.59] or moderately high/severe [boys-OR 4.58; CI 2.88–7.29 and girls-OR 4.18; CI 3.1–5.63] depressive symptoms had significantly higher odds of deliberate self-harm.

**Conclusions:**

Internet access, parental abuse, involvement in fights, and depressive symptoms emerged as significant predictors of deliberate self-harm among adolescent boys and girls. Results suggest that an early identification of the predictors and intervention might prevent deliberate self-harm among adolescents. Since parents play a major role in the lives and development of adolescents, it is highly recommended that they initiate open and supportive communication with their children.

## Background

Inflicted to self-harm (ISH) or Deliberate self-harm (DSH) is ‘a behavior that emerges among children and adolescents in which a child/adolescent commits an act with the purpose of physically or psychologically harming himself/herself with or without a real intent of suicide’ [1]. Self-inflicted or deliberate self-harm is noticed across different age groups and socio-economic groups within the society on different scales [1–3]. However, it is more frequent among the adolescent population. Furthermore, the causes and severity of self-inflicted harm among adolescents varies within and between countries, and across different socio-economic settings [4, 5]. While there are many forms of self-inflicted harms like carving, scratching, branding, marking, picking or pulling skin or hair, burning, cutting, biting, hitting or excessive body piercing [1] that might not cause serious health outcome, there are other forms which are dangerous in nature and can cause serious injuries and deaths.

In case of India, very little attention has been paid by family members, the community, and even the government to those children and adolescents who have problems with self-infliction or associated risk behaviors. Adolescents surrounded by cultural and social situations determine their behavior in terms of self-inflicted harm, aggression, anxiety, substance abuse, and socio-psychological behavior [6–8]. High socio-economic inequality in India has led to an increase in self-inflicted harm among children and adolescents. For instance, adolescents belonging to lower social groups are more prone to deliberate self-harm than their counterparts who belong to higher social groups [9]. Furthermore, social and cultural norms play an enormous role in defining child and adolescent’s health and wellbeing in India, and it further molds behavioral change among them [8, 10].

Self-inflicted harm is a complex behavioral phenomenon and a symptom that results from a variety of factors [2, 11]. Adolescents or children who have difficulty in talking about their feelings may show their emotional tension or stress, aggressive behavior, physical discomfort, pain, and low self-esteem with self-injurious behaviors and many more [12]. For example, aggressive behavior is one such risk factor that causes self inflicted harm [13–15]. Studies have established a relationship between self-inflicted harm and aggressive behavior among children and adolescents [3, 16].

Inflicted self-harm (ISH) has been recognized worldwide as a major public health issue, with a severe and serious impact on the individual, their family, community, and the healthcare system per se [17]. There are cross-sectional studies that show a number of factors that are responsible for self-harm among adolescents [3, 12, 14, 18]. Adolescents who are inflicted with self-harm are more prone to suicide or suicidal ideation [3, 11, 19]. Researchers have identified various conducive factors such as peers, school, family, religious and others that affect suicidal ideation and self-inflicted harm in adolescents [1, 14, 18]. Recently research has also linked the aggravated effects of bullying including cyberbullying to inflicted self-harm and aggressive behaviour [20].

Self inflicted harm as a behavior is an interaction of multi-causal problems that often emerge among the young and adolescent population [21, 22]. Worldwide, a high prevalence of self-inflicted behavior is found among the young and adolescent population [23–25]. Additionally, it is caused at multiple levels like at the individual, household and community level [25–27]. Furthermore, the most significant factors that determine self-inflicted harm among adolescents are socio-psychological, economic and family-related problems [27–29]. In case of India, multiple causes invoke self-inflicted harm among adolescents like physical abuse in childhood, substance abuse such as alcohol and tobacco consumption, negative peer influence, family-violence, academic disturbance, aggressive behaviour, psychological problems, attention deficit-hyperactivity disorder, and loneliness [25]. Though it is highly prevalent in high income countries, however, recent trends have shown that the low and middle income countries including India are also facing an emergence of self-inflicted harm among children and adolescents that disrupt their health and social well-being [30, 31].

A survey of the recent literature show various factors that are associated with the occurrence of self inflicted harm among children and adolescents. For example, it can be due to genetic or hereditary reasons [22, 23, 32]. Again, some studies show it is not only hereditary in nature but there are other factors responsible for the emergence of such behavior among adolescents [27, 33–36]. These factors include previous aggression and violent behavior, exposure to violence in-home or community, use of drugs and alcohol, the combination of stressful socio-economic and family factors and further being a victim of bullying and abuse that lead to high aggression among children and adolescents [21, 23, 29, 37–39]. The risk factors for self inflicted harm can be classified as psychosocial conditions of the environment, individual-specific characteristics of the population and their interactions with several associated determinants [40].

The occurrence of self inflicted harm among adolescents is a self-destructive phenomenon and has a huge socio-psycho and health impact on individuals, families, friends and even communities [23, 39, 41]. This can be a huge problem for children and adolescents with both normal development and those with psychosocial disturbances [23, 24, 34]. Children and adolescents with such behaviour can also be destructive in nature and that includes a wide range of behaviors: explosive temper tantrums, physical aggression, fighting, threats or attempts to hurt others (including thoughts of wanting to kill others), use of weapons, cruelty toward animals, fire setting, intentional destruction of property and vandalism [27, 34, 39, 42, 43]. Further, it is also linked to suicidal ideation or behavior among them [25, 44]. Among the children and adolescent population, there are various causes that make them susceptible to self inflicted harm such as poor relationship skills, substance abuse, underlying health causes and stress or frustration [22, 34, 36]. Sometimes adolescent population also face the problem of loneliness, has difficulty with social interaction and interpersonal communication that leads to impulsiveness and aggressiveness [24, 27, 29]. Numerous research studies and clinical trials have concluded on different aspects of child and adolescent population such as health behavior, mental and psychological issues and anxiety, depression, self-inflicted harm [2, 3, 14], and suicidal tendency and found a complex interaction of factors that led to an increased risk of violent and aggressive and self-inflicted harm behavior in children and adolescents [24, 25, 28, 33, 40].

On the basis of previous literature, which has underlined the correlation between adolescents’ hereditary, psychological problems and drug abuse with their self-inflicted harm, however, a dearth of literature exist on their socio-economic characteristics as risk factors of their self-inflicted harm. Therefore, to understand the nuance of self-inflicted harm among adolescent population, this paper assesses its risk factors across different socio-economic settings in the two Indian states of Bihar & Uttar Pradesh. The paper hypotheses that there is no such self-inflicted harm among adolescents across different socio-economic settings.

## Methods

### Data description and sample selection procedure

Data for this study was obtained from Understanding the Lives of Adolescents and Young Adults (UDAYA) project survey. The survey was conducted in the two Indian states namely, Uttar Pradesh and Bihar in 2016 by Population Council under the guidance of Ministry of Health and Family Welfare, Government of India. The survey collected detailed information on family, media, community environment, assets acquired in adolescence, and quality of transitions to young adulthood indicators. The UDAYA survey adopted a multi-stage systematic sampling design to provide the estimates for states as a whole as well as urban and rural areas of the states [45]. A total of 150 primary sampling units (PSUs)—villages in rural areas and census wards in urban areas had visited in both states in order to conduct interviews in the required number of households. The 150 PSUs were further divided equally into rural and urban areas, that is, 75 for rural respondents and 75 for urban respondents. The 2011 census list of villages and wards (each consisting of several census enumeration blocks [CEBs] of 100–200 households) served as the sampling frame for the selection of villages and wards in rural and urban areas, respectively. This list was stratified using four variables, namely, region, village/ward size, proportion of the population belonging to scheduled castes and scheduled tribes, and female literacy. The household sample in rural areas was selected in three stages, while in urban areas it was selected in four stages. In rural areas, villages were first selected systematically from the stratified list as described above, with selection probability proportional to size (PPS). In urban areas, 75 wards were first selected systematically with probability proportional to size, and within each selected ward, CEBs were then arranged by their administrative number and one CEB was randomly selected. Several CEBs adjacent to the selected CEB were merged to ensure at least 500 households for listing.

The sample size for Uttar Pradesh and Bihar was 10,350 and 10,350 adolescents aged 10–19 years, respectively. UDAYA was designed to provide estimates for the state as a whole as well as for the urban and rural areas of the state for each of the five categories of respondents. The required sample for each sub-group of adolescents was determined at 920 younger boys, 2,350 older boys, 630 younger girls, 3,750 older girls, and 2,700 married girls in both states. The analysis was done only for unmarried adolescents. Therefore, the effective sample size for this study was 5,969 adolescent boys and 9,419 girls aged 10–19 years.

## Questionnaire and measures

### Outcome variables

The outcome variable was deliberate self-harm among adolescents. The variable was generated using three questions: (a) During the past 12 months, when you are agitated, angry or sad, have you ever cut/bitten yourself? If yes, would you say 1–2 times, 3–4 times, 5 + times? (b) During the past 12 months, when you are agitated, angry or sad have you ever pulled your own hair? If yes, would you say 1–2 times, 3–4 times, 5 + times? (c) During the past 12 months, when you are agitated, angry or sad have you ever banged or hit yourself? If yes, would you say 1–2 times, 3–4 times, 5 + times? All the three variables were coded as 0 if deliberate self-harm and 1 if deliberate self-harm was done (includes 1–2 times, 3–4 times, 5 + times). Then a new variable was formed with a scale of 0–3 (summation of all three variables); wherein 0 represents no deliberate self-harm and 1–3 was coded as “1” representing the deliberate self-harm. Hence, a dichotomous (*outcome- deliberate self-harm*) variable was formed using these three questions i.e., the respondent was categorized as having deliberate self-harm if he/she responded as yes in either of the three question and no, otherwise.

### Explanatory variable

*Individual factors*

1. Age which was in years was recoded as early adolescents [10–14] and late adolescents [15–19]
2. Current schooling status was recoded as never attended, dropout and currently attending.
3. Working status was recoded as not working and working.
4. Media exposure was recoded as no exposure, rare exposure and frequent exposure.
5. Access to internet was recoded as no and yes.
6. Parental abuse means if the adolescent was physically hurt by either father or mother was coded as no, yes and parents do not co-reside.
7. Involvement in fights was coded as no and yes.
8. Substance use was coded as no and yes. Adolescent who consumed either tobacco products or alcohol were considered to be consuming substance.
9. Depressive symptoms were assessed by asking nine questions from the respondents, the respondent was asked about the symptoms for past two weeks only. The questions included, a. had trouble falling asleep or sleeping too much, b. feeling tired or having little energy, c. poor appetite or eating too much, d. trouble concentrating on things, e. had little interest or pleasure in doing things f. feeling down, depressed or hopeless, g. feeling bad about yourself, h. been moving or speaking slowly, i. had thoughts that respondent would be better off dead. All the above questions were asked on a scale of four i.e., 0 “not at all”, 1 “less than one week”, 2 “one week or more” and 3 “nearly every day”. The scale of 27 points was then generated using egen command in STATA 14. (Cronbach alpha: 0.86) [46]. The variable was then recoded into three categories I.e., a. Mild (0–9), b. Moderate (10–14) and c. Severe (15–27). Mild includes minimal and mild; moderate include moderate only and severe include moderately severe and severe (Cronbach Alpha: 0.90). The categories were redefined for analytical purpose.
*Household factors*
10. Caste was recoded as Scheduled Caste and Scheduled Tribe (SC/ST) and non-SC/ST. The Scheduled Caste include a group of population which is socially segregated and financially/economically by their low status as per Hindu caste hierarchy. The Scheduled Castes (SCs) and Scheduled Tribes (STs) are among the most disadvantaged socio-economic groups in India [47, 48].
11. Religion was recoded as Hindu and non-Hindu. The category of non-Hindu was recoded because the frequency of other religions was very low, and therefore for analytical purpose the recoding was done in the respective manner.
12. Wealth index was recoded as poorest, poorer, middle, richer and richest. The survey measured household economic status, using a wealth index composed of household asset data on ownership of selected durable goods, including means of transportation, as well as data on access to a number of amenities. The wealth index was constructed by allocating the following scores to a households reported assets or amenities. Then using the scores were divided into five quintiles.
13. Residence was available in data as urban and rural.
14. Data was available for two states i.e., Uttar Pradesh and Bihar. As the survey was conducted in these two states only.

### Statistical analysis

Descriptive statistics along with bivariate analysis was done to examine the preliminary results. For analyzing the association between the binary outcome variable and other explanatory variables binary logistic regression method was used. The outcome variable was deliberate self-harm among adolescents aged 10–19 years. The data was analyzed separately for adolescent boys and adolescent girls.

Equation for logistic regression

$$\ln \left( {\frac{\pi }{1 - \pi }} \right) = \alpha + \beta_{1} X_{1} + \beta_{2} X_{2} + \beta_{3} X_{3} \ldots \beta_{n} X_{n}$$

where $\beta_{0} , \ldots ,\beta_{M}$ are regression coefficients indicating the relative effect of a particular explanatory variable on the outcome. These coefficients change as per the context in the analysis in the study.

## Results

### Socio-demographic characteristics of study population (Table 1)

**Table 1:** Socio-demographic profile of adolescents aged 10–19 years

Variables	Adolescent boys		Adolescent girls	
	Sample	Percentage	Sample	Percentage
Age group				
Early adolescents	2084	34.9	1653	17.6
Late adolescents	3885	65.1	7766	82.5
Current schooling status				
Never attended	190	3.2	639	6.8
Dropout	1092	18.3	2559	27.2
Currently attending	4687	78.5	6221	66.1
Working status				
Not working	4377	73.3	7582	80.5
Working	1592	26.7	1837	19.5
Media exposure				
No exposure	335	5.6	1345	14.3
Rare	1078	18.1	2539	27.0
Frequent	4555	76.3	5535	58.8
Internet access				
No	4246	71.1	8673	92.1
Yes	1723	28.9	746	7.9
Parental abuse				
No	2329	39.0	5885	62.5
Yes	3480	58.3	3276	34.8
Parent don't co-reside	160	2.7	258	2.7
Involved in fights				
No	4002	67.0	8011	85.1
Yes	1967	33.0	1408	15.0
Substance use				
No	4988	83.6	9271	98.4
Yes	981	16.4	148	1.6
Depressive symptoms				
Minimal/mild	5541	92.8	8105	86.1
Moderate	255	4.3	621	6.6
Moderately high/ severe	173	2.9	692	7.4
Caste				
SC/ST	1605	26.9	2241	23.8
Non-SC/ST	4364	73.1	7178	76.2
Religion				
Hindu	5024	84.2	7234	76.8
Non-Hindu	945	15.8	2185	23.2
Wealth index				
Poorest	704	11.8	1213	12.9
Poorer	1193	20.0	1666	17.7
Middle	1374	23.0	1966	20.9
Richer	1391	23.3	2315	24.6
Richest	1308	21.9	2259	24.0
Residence				
Urban	1030	17.3	1625	17.3
Rural	4939	82.7	7794	82.7
State				
Uttar Pradesh	4069	68.2	6637	70.5
Bihar	1900	31.8	2782	29.5
Total	5969	100.0	9419	100.0

SC/ST: Scheduled Caste/Scheduled Tribe

A higher proportion of study participants were late adolescents (boys-65.1% and girls-82.5%) and currently attending school (boys-78.5% and girls-66.1%). Moreover, about one-fourth of adolescent boys and one in every five girls were working. Around three-fourth of boys and 59 per cent of girls had frequent media exposure. Three in every ten boy and only eight per cent of girls had internet access. About 58 per cent of boys and 35 per cent of girls experienced parental abuse, one-third of boys and 15 per cent of girls involved in fights, and 16 per cent of boys and only two per cent of girls were using substance use. Nearly three per cent of adolescent boys and seven per cent of girls had moderately high/severe depressive symptoms.

### Deliberate self-harm among adolescents by background characteristics (Table 2)

**Table 2:** Percentage distribution of deliberate self-harm among adolescents by background characteristics

Background characteristics	Adolescent boys		Adolescent girls	
	Deliberate self-harm behavior	p < 0.05	Deliberate self-harm behavior	p < 0.05
Age group				*
Early adolescents	5.3		6.1	
Late adolescents	4.1		2.5	
Current schooling status		*		
Never attended	6.8		2.9	
Dropout	4.7		3.2	
Currently attending	4.4		3.2	
Working status				
Not working	4.4		3.3	
Working	4.9		2.6	
Media Exposure				
No exposure	6.0		3.2	
Rare	3.7		3.4	
Frequent	4.6		3.1	
Internet access				*
No	4.8		3.0	
Yes	3.9		5.5	
Parental abuse		*		*
No	2.6		2.5	
Yes	5.9		4.5	
Parent don't co-reside	2.4		1.6	
Involved in fights		*		*
No	2.7		2.3	
Yes	8.3		8.0	
Substance use		*		*
No	4.2		3.1	
Yes	6.1		5.5	
Depressive symptoms		*		*
Minimal/mild	3.8		2.7	
Moderate	12.2		5.2	
Moderately high/ severe	15.1		6.9	
Caste				
SC/ST	4.1		3.3	
Non-SC/ST	4.7		3.1	
Religion		*		*
Hindu	4.3		2.8	
Non-Hindu	5.9		4.3	
Wealth index				
Poorest	6.1		2.3	
Poorer	3.7		3.4	
Middle	4.2		3.0	
Richer	4.2		3.3	
Richest	5.1		3.4	
Residence				*
Urban	4.7		4.2	
Rural	4.5		2.9	
State		*		*
Uttar Pradesh	4.7		3.4	
Bihar	4.2		2.6	
Total	4.5		3.2	

SC/ST: Scheduled Caste/Scheduled Tribe

About 4.5% and 3.2% of adolescent boys and adolescent girls had deliberate self-harm behavior respectively. It was found that deliberate self-harm behavior was significantly more prevalent among early adolescent girls (6.1%) compared to late adolescent girls (2.5%). The result was similar for adolescent boys also, but it was not significant. Interestingly, adolescent boys who never attended school had significantly higher prevalence of deliberate self-harm behavior (6.8%). The prevalence of deliberate self-harm behavior was higher among adolescent girls who had internet access (5.5%) than who did not have internet access (3%). Adolescents who experienced parental abuse had higher prevalence of deliberate self-harm behavior (boys-5.9% and girls-4.5%) compared to those who did not faced it (boys-2.6% and girls-2.5%). Similarly, adolescents who were involved in fights also had more deliberate self-harm behavior (boys-8.3% and girls-8%) than those who were not involved. The prevalence of deliberate self-harm behavior was significantly higher among adolescents who were using substance use (boys-6.1% and girls-5.5%) compared to those who were not using it. Interestingly, adolescents who had moderately high/severe depressive symptoms, had significantly higher prevalence of deliberate self-harm behavior (boys-15.1% and girls-6.9%). The prevalence of deliberate self-harm behavior was more among girls who lived in urban areas (4.2%).

### Estimates from logistic regression analysis by background characteristics (Table 3)

**Table 3:** Logistic regression estimates for deliberate self-harm among adolescents aged 10–19 years by background characteristics

Background characteristics	Adolescent boys	Adolescent girls
	OR (95% CI)	OR (95% CI)
Age group		
Early adolescents	Ref	Ref
Late adolescents	1.20 (0.83, 1.75)	0.75 (0.54, 1.03)
Current schooling status		
Never attended	Ref	Ref
Dropout	1.01 (0.5, 2.03)	1.08 (0.64, 1.83)
Currently attending	1.01 (0.5, 2.03)	1.03 (0.62, 1.73)
Working status		
Not working	Ref	Ref
Working	1.12 (0.81, 1.56)	1.0 (0.73, 1.36)
Media Exposure		
No exposure	Ref	Ref
Rare	0.65 (0.34, 1.25)	1.02 (0.65, 1.6)
Frequent	0.67 (0.37, 1.23)	0.99 (0.64, 1.52)
Internet access		
No	Ref	Ref
Yes	0.98 (0.7, 1.36)	1.50* (1.05, 2.16)
Parental abuse		
No	Ref	Ref
Yes	1.49* (1.11, 2)	1.61* (1.27, 2.04)
Parent don't co-reside	1.08 (0.52, 2.22)	0.84 (0.4, 1.74)
Involved in fights		
No	Ref	Ref
Yes	2.94* (2.24, 3.85)	2.22* (1.71, 2.87)
Substance use		
No	Ref	Ref
Yes	1.26 (0.89, 1.77)	1.55 (0.85, 2.82)
Depressive symptoms		
Minimal/mild	Ref	Ref
Moderate	2.10* (1.29, 3.4)	2.50* (1.74, 3.59)
Moderately high/ severe	4.58* (2.88, 7.29)	4.18* (3.1, 5.63)
Caste		
SC/ST	Ref	Ref
Non-SC/ST	1.35 (0.96, 1.89)	0.87 (0.65, 1.17)
Religion		
Hindu	Ref	Ref
Non-Hindu	1.17 (0.83, 1.63)	1.15 (0.87, 1.52)
Wealth index		
Poorest	Ref	Ref
Poorer	0.77 (0.47, 1.27)	1.31 (0.8, 2.15)
Middle	0.78 (0.48, 1.26)	1.24 (0.76, 2.02)
Richer	0.76 (0.47, 1.24)	1.34 (0.83, 2.17)
Richest	0.82 (0.49, 1.37)	1.51 (0.9, 2.51)
Residence		
Urban	Ref	Ref
Rural	0.97 (0.72, 1.31)	0.78 (0.60, 1.02)
State		
Uttar Pradesh	Ref	Ref
Bihar	0.80 (0.61, 1.05)	0.70* (0.55, 0.89)

*p < 0.05; OR: Odds Ratio; CI: Confidence Interval; Ref: Reference; SC/ST: Scheduled Caste/Scheduled Tribe

Results revealed that the odds of deliberate self-harm behavior were 50 per cent more likely among adolescent girls who had internet access [OR 1.50; CI 1.05–2.16] compared to those who did not have it. The likelihood of deliberate self-harm behavior was 49 per cent and 61 per cent significantly more likely among adolescent boys [OR 1.49; CI 1.11–2.0] and girls [OR 1.61; CI 1.27–2.04] who experienced parental abuse respectively than those who did not experience it. Moreover, adolescent boys [OR 2.94; CI 2.24–3.85] and girls [OR 2.22; CI 1.71–2.87] who were involved in fights were 2.94 times and 2.22 times more likely to have deliberate self-harm behavior respectively compared to those who were not involved in fights. The likelihood of deliberate self-harm behavior was more likely among adolescents who were using substance use compared to those who were not using it. Though, the result was not significant. With reference to minimal/mild depressive symptoms, adolescents who had moderate [boys-OR 2.10; CI 1.29–3.4 and girls-OR 2.50; CI 1.774–3.59] or moderately high/severe [boys-OR 4.58; CI 2.88–7.29 and girls-OR 4.18; CI 3.1–5.63] depressive symptoms had significantly higher odds of deliberate self-harm behavior.

## Discussion

The present research attempted to analyze various risk factors of deliberate self-harm among adolescents in Bihar and Uttar Pradesh by utilizing data from Understanding the Lives of Adolescents and Young Adults (UDAYA) project survey. Results from statistical analysis indicate that Internet access, parental abuse, involvement in fights, and depressive symptoms to be significant predictors of deliberate self-harm among adolescent boys and girls. Accepting our working hypothesis, the study results exhibit that there is no deliberate self-harm behavior among adolescents across different socio-economic settings. Nevertheless, there is value in the findings because it provides significant insights into the risk factors that support deliberate self harm among adolescent boys and girls.

Results of both bivariate and multivariate analysis indicate that the likelihood of deliberate self-harm is high among adolescent girls who have Internet access. A study by Mitchell & Ybarra, 2007, though not focusing on the gender aspect, emphasized that adolescents who spend a substantial amount of time online and have access to Internet are the ones who frequently self-harm themselves than those who do not have access to it [49]. Evidence from recent studies indicate that adolescents who have thoughts of deliberate self-harm and self-harm behaviors place a high value on accessing Internet resources, including those on social media [50], prefer the Internet as a means to retrieve Non-suicidal self-injury (NSSI) resources [51]. Moreover, a study conducted on adolescents in Italy revealed adolescents with Internet use experience lower quality relationships with their parents and have more individual difficulties [52]. In fact, adolescents with Internet addiction are more likely to have aggressive behaviors [53] and since aggressive behaviour is a cause of self-harm [6–8] one cannot deny the influence of it. Aggressive behavior due to internet overuse like addiction to play games, [54], and problematic relationships with parents and peers [33] can affect deliberate self-harm.

Most of the time moderate to harsh physical punishment is adopted by parents to discipline their children. In a study on sample of mothers from Brazil, Chile, Egypt, India, Philippines, and the United States, it was found that nearly all parents used nonviolent and verbal or psychological punishment to discipline their children. However, in India rates of slapping on the face or head exceeded spanking as a discipline practice [55]. Likewise, in Chinese culture parents adopt harsh discipline to motivate their children to achieve higher academic grades, socially appropriate behaviors, and positive psychological adjustment at school [56, 57]. These methods of harsh physical disciplinehave often triggered long term detrimental influences like deliberate self-harm among adolescents [58–60]. The results of the present study are also in agreement with the previous studies that indicate adolescent boys and girls who have experienced parental abuse have higher likelihood of deliberate self-harm. Reasons for deliberate self-harm due to negative parenting could range from a compensatory strategy to regulate distressing emotions [59] and fractured identity [61] to having poorer social self-worth and self-competence [58].

Prior research indicate a positive relationship with deliberate self-harm among adolescents and depressive symptoms [5, 62, 63]. A study on Finnish adolescents aged 13–18 years found out depression to be an independent risk factor of self harm [64]. A recently published qualitative study showed that adolescents who self-harm feel isolated and express distress emotions [4]. Findings from another study indicated that adolescents suffering from mental illness thought it to be a stigma and therefore, could not seek help. The same study concluded that adolescents considered family, friends and school as the main sources of support in preventing suicidal behaviour, and more pertinent than external helping agencies [65].

## Limitation of the study

Since the methodology used is cross-sectional, it was impossible to draw conclusions regarding causal relationships between the different predictors and the outcome variable, deliberate self-harm among the adolescents. Also, the results of the present study focused only on two states of India i.e., Bihar and Uttar Pradesh. Therefore, one needs to be cautious while interpreting the results for the entire country. Additionally, although our findings highlighted the potential risk factors of deliberate self-harm among the adolescents, future research in the form of qualitative studies on an in-depth understanding of what triggers the urge to self-harm among adolescents is required.

## Conclusion

The results of the present study are useful in identifying adolescents who are at-risk of deliberate self-harm. Programs aimed at preventing deliberate self-harm should consider the role of parents. It is highly recommended that parents initiate open and supportive communication with their children so that adolescents do not rely on non-credible sources on the internet. Efforts to facilitate adolescents access to credible Internet resources are also needed. Parents of adolescents who self-harm should be counselled on the long term and harmful effects of parental physical abuse. Promotion of both positive parenting skills and mental health may build resilience to self-harm thoughts and acts among adolescents.

## Data Availability

The study utilizes a secondary source of data that is freely available in the public domain through, https://dataverse.harvard.edu/dataset.xhtml?persistentId=doi:10.7910/DVN/ZJPKW5.

## Abbreviations

OR: Odds Ratio
CI: Confidence Interval
UDAYA: Understanding the Lives of Adolescents and Young Adults
PTSD: Post-Traumatic Stress Disorder
SC/ST: Scheduled Caste/Scheduled Tribe
